# An Update on Advances in COVID-19 Laboratory Diagnosis and Testing Guidelines in India

**DOI:** 10.3389/fpubh.2021.568603

**Published:** 2021-03-04

**Authors:** K. S. Rajesh Kumar, Suhail Sayeed Mufti, Vinu Sarathy, Diganta Hazarika, Radheshyam Naik

**Affiliations:** ^1^Department of Translational Medicine and Therapeutics, HealthCare Global Enterprises Ltd. (HCG), Bangalore, India; ^2^Department of Medical Oncology, Hematology and BMT, HealthCare Global Enterprises Ltd. (HCG), Bangalore, India

**Keywords:** COVID-19, RT-PCR, guidelines, laboratory diagnosis, molecular testing

## Abstract

The declaration of COVID-19 as a global pandemic has warranted the urgent need for technologies and tools to be deployed for confirming diagnosis of suspected cases. Diagnostic testing for COVID-19 is critical for understanding epidemiology, contract-tracing, case management, and to repress the transmission of the SARS-CoV-2. Currently, the Nucleic Acid Amplification Test (NAAT)-based RT-PCR technique is a gold standard test used for routine diagnosis of COVID-19 infection. While there are many commercially available RT-PCR assay kits available in the market, selection of highly sensitive, specific, and validated assays is most crucial for the accurate diagnosis of COVID-19 infection. Laboratory diagnosis of SARS-CoV-2 is extremely important in the disease and outbreak management. Development of rapid point of care tests with better sensitivity and specificity is the critical need of the hour as this will help accurate diagnosis and aid in containing the spread of SARS-CoV-2 infection. Early detection of viral infection greatly enhances implementation of specific public health intervention, such as infection control, environmental decontamination, and the closure of specific high-risk zones. Large-scale sequencing of SARS-CoV-2 genome isolated from affected populations across the world needs to be carried to monitor mutations that might affect performance of molecular tests. Creation of genome repositories and open-source genetic databases for use by global researchers is clearly the way forward to manage COVID-19 outbreak and accelerate vaccine development. This review summarizes various molecular diagnostics methods, technical guidelines, and advanced testing strategies adopted in India for laboratory diagnosis of COVID-19.

## Introduction

On March 12, 2020, the World Health Organization (WHO) declared the novel coronavirus disease 2019 (COVID-19) caused by the Severe Acute Respiratory Syndrome Coronavirus 2 (SARS-CoV-2) as global pandemic. According to the WHO data on May 14, 2020, there have been 4,218,212 confirmed cases of COVID-19, including 290,242 deaths reported globally and 74,281 confirmed cases reported from India (1). The declaration of COVID-19 as a global pandemic has warranted the urgent need for technologies and tools to be deployed for confirming diagnosis of suspected cases. Diagnostic testing for COVID-19 is critical for understanding epidemiology, contract-tracing, case management, and to repress the transmission of the SARS-CoV-2. Hence, there is an urgent need for deployment of rapid, highly specific and ultra-sensitive molecular diagnostic tests. Most importantly, accurate and rapid diagnosis of SARS-CoV-2 infection will help to identify, isolate, and treat the patients in order to minimize risk of public contamination and drastically reduce the mortality rates. As per the existing guidelines, the clinicians should work with their local and state health departments to coordinate laboratory diagnosis of COVID-19 through government laboratories or work with commercial clinical laboratories (2).

Currently, WHO recommends for laboratory diagnosis COVID-19 based on detection of unique sequences of virus RNA by Nucleic Acid Amplification Test (NAAT) such as real-time reverse-transcriptase polymerase chain reaction (rRT-PCR). Similarly, the Indian Council of Medical Research (ICMR) also recommended use of rRT-PCR-based tests approved by the US FDA EUA/CE-IVD approved kits, under intimation to Drug Controller General of India (DCGI) and Ministry of Health and Family Welfare (MoH&FW). The FDA EUA/CE-IVD approved RT-PCR kits are highly specific and detects the presence or absence of SARS-CoV-2 viral nucleic acid and thus directly confirms viral infection in a human sample. The RT-PCR test is the current gold standard diagnostic method for the diagnosis of COVID-19. Other molecular methods such as virus antigen or serological antibody testing are currently recommended for use only in research settings and not in clinical decision-making (3).

### Structure of SARS-CoV-2

SARS-CoV-2 is a large positive-sense single-stranded ribonucleic acid (RNA) virus belonging to the family Coronaviridae.

**Figure 1:**
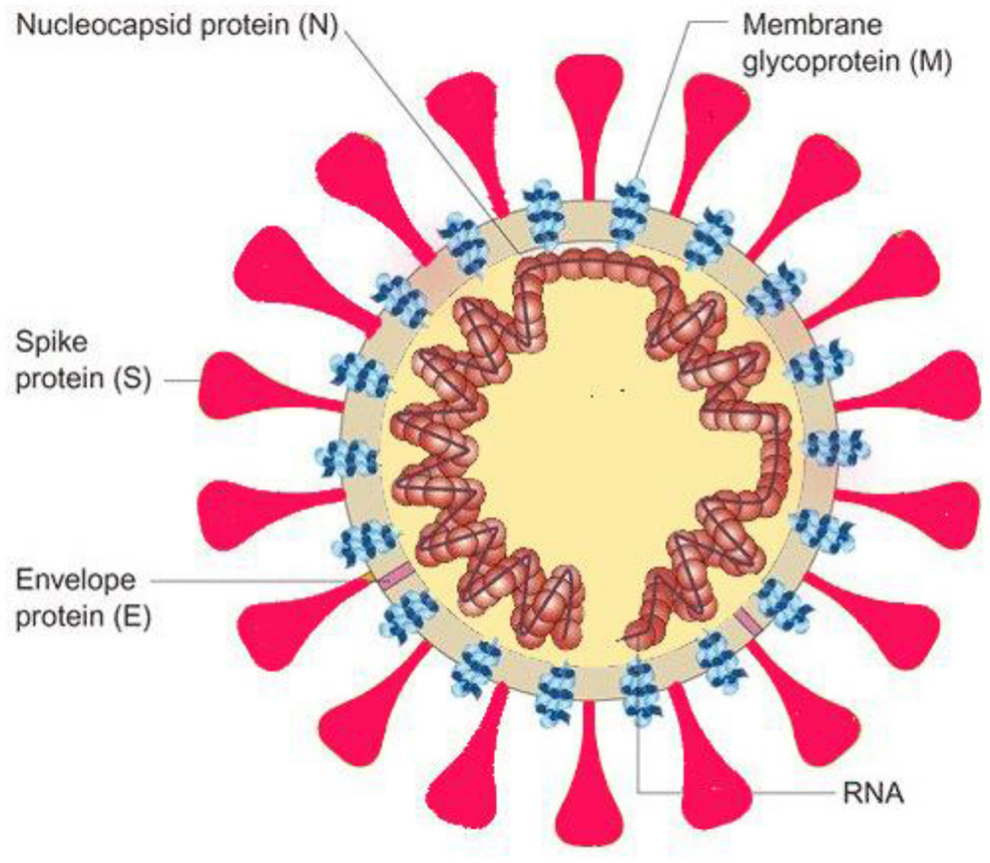
Illustration of viral structure with its structural viral proteins (4).

The SARS-CoV-2 genome is approximately 30,000 nucleotides in length and encodes several proteins including an RNA-dependent RNA polymerase (RdRP) and four structural proteins viz., nucleocapsid protein (N), spike protein (S), envelope protein (E), and membrane protein (M) (Figure 1). RdRP helps in maintaining fidelity of viral genome by acting in conjunction with nonstructural proteins, and the spike protein (S) plays an important role in transmission of the virus by functioning in receptor binding and membrane fusion in the host (5). The S gene of the SARS-CoV-2-encoding spike protein is found to have <75% nucleotide sequence similarity when compared to other SARS-related coronaviruses. The E, M, and N structural proteins are more conserved than the spike protein and are essential for general function of coronavirus (6, 7).

### Viral Replication

Coronaviruses have complex machinery comprising of multisubunits for replication and transcription. Around 8–10 open reading frames (ORFs) are found in most of the coronaviruses, among them ORF1a and ORF1b are important in SARS-CoV-2. ORF1a and ORF1b are translated into polyprotein 1a (pp1a) and pp1ab. RNA-dependent RNA polymerase enzyme (RdRp) is produced as a result of viral protease cleavage of pp1a and pp1ab polyproteins. RdRp, also known as nsp12, plays a predominant role in the replication and transcription cycle of COVID-19 virus and hence is being considered as primary target for antiviral inhibitors like remdesivir (8, 9). Coronaviruses generate 6–9 subgenomic mRNAs (sgmRNAs) during replication, and structural and accessory proteins are translated from this sgmRNAs (10).

## Sample Requirement for COVID-19 Testing

Appropriate sample collection is the most important step in the laboratory diagnosis of any infectious disease. Improper specimen collection may contribute to false negative test results. Table 1 summarizes different types of samples used for COVID-19 testing as recommended by ICMR (11). The guideline mandates clinical sample collection by trained laboratory personnel/health-care workers in the presence of a clinician. Generally, samples for COVID-19 diagnosis are collected from two major sources: upper respiratory tract and lower respiratory tract. Upper respiratory tract specimens are collected by nasopharyngeal (NP) swab or the oropharyngeal (OP) swab, whereas the bronchoalveolar lavage, tracheal aspirate, or sputum will be collected from the lower respiratory tract. ICMR has created a comprehensive Specimen Referral Form for COVID-19 for use by all specimen collection centers and testing labs (12).

**Table 1:** Description of samples used for COVID-19 detection.

**Specimen type**	**Collection materials**	**Transport to laboratory**	**Storage till testing**	**Suitable for Tests**
Nasopharyngeal and oropharyngeal swab	Flocked swabs	4°C	≤5 days: 4°C > 5 days: −70°C	RT-PCR, Rapid Diagnostic Test
Bronchoalveolar lavage	Sterile container	4°C	≤48 h: 4°C > 48 h: −70°C	RT-PCR, Rapid Diagnostic Test
Tracheal aspirate, nasopharyngeal aspirate, or nasal wash	Sterile container	4°C	≤48 h: 4°C >48 h: −70°C	RT-PCR, Rapid Diagnostic Test
Sputum	Sterile container	4°C	≤48 h: 4°C > 48 h: −70°C	RT-PCR, Rapid Diagnostic Test
Whole blood (5 ml)	EDTA Vial	4°C	≤5 days: 4°C	ELISA, Immunodiagnostics

### Nasopharyngeal Swabs

The NP specimen is a vital and sensitive sample to test the SARS-CoV-2 virus. As suggested by the Centers for Disease Control and Prevention (CDC), it is highly recommended to collect only the NP swab, although OP swabs remain an acceptable specimen type. In the case that both NP and OP swabs are collected, they should be combined in a single tube to maximize sample load and test sensitivity. Synthetic fiber swabs with plastic shafts are recommended for NP and OP samples collection. Calcium alginate or wooden-shaft swabs are not recommended because they might inactivate the virus and could provide a negative result. NP and OP samples should be placed immediately into an appropriate sterile medium or saline for proper transportation.

### Oropharyngeal Swabs

The OP swab is another important specimen recommended by the WHO and CDC to detect SARS-CoV-2 infection. The OP swab is collected from the posterior pharynx region, avoiding contact with the tongue.

### Bronchoalveolar Lavage and Tracheal Aspirate

Collection of the bronchoalveolar lavage (BAL) and tracheal aspirate is recommended only in severely ill and hospitalized patients.

### Sputum

Sputum is collected from patients who have severe coughing symptoms. Sputum is collected by asking patients to expectorate deep-cough sputum directly straight into a sterile, leak-proof, screw-cap sputum collection cup or sterile dry container.

### Other Samples

Blood and stool samples are also used in diagnosis of infection since SARS-CoV-2 is known to present in blood and stool. A small study reported the presence of SARS-CoV-2 in anal or oral swabs of patients in the Hubei Province (13). However, the utility of these samples remains unclear, because the data on viral shedding post-infection is still preliminary.

### Nucleic Acid Amplification Test (NAAT)

NAAT-based RT-PCR technique is currently a gold standard test used for routine confirmation of cases of COVID-19. The viral genes targeted in the RT-PCR test so far include the N, E, S, and RdRP genes. According to the WHO guidelines, in order to confirm the positive diagnosis, a validated RT-PCR assay targeting a minimum of two regions on the SARS-CoV-2 genome must be chosen, with one being specific for SARS-CoV-2. Alternatively, the test can include a primer specific for betacoronavirus and presence of SARS-CoV-2 must be confirmed by sequencing partial or whole genome of the virus (14).

ICMR recommends use of commercial RT-PCR-based tests approved by the US FDA EUA/CE-IVD approved kits, under intimation to DCGI and MoH&FW under Biosafety 2 level (BSL-2) conditions and with appropriate biosafety precautions. The commercial testing kits also need to be validated by ICMR before by mass testing. These tests are validated at National Institute of Virology (NIV), Pune, and 14 other validation centers approved by ICMR. As of May 25, 2020, 83 commercial RT-PCR kits have been validated by ICMR validation centers, and 33 kits were found to be satisfactory for testing (15). Conventional PCR assays, in-house developed RT-PCR assays, and antigen/antibody testing are not recommended for clinical diagnosis of COVID-19 (16). ICMR NIV has issued technical guidelines for testing of suspected human sample by RT-PCR assay. According to this guideline, samples should be first tested in the E gene screening assay and later tested in the RdRp, ORF1b, and N gene confirmatory assays. These assays are highly sensitive and specific and do not cross-react with other coronavirus or human clinical samples that contain respiratory viruses. Table 2 summarizes examples of primer and probe details used in screening and confirmatory assay (17, 18).

**Table 2:** Details of primers and probes for screening and confirmatory assay.

	**Assay**	**Oligonucleotide ID**	**Sequence (5^**′**^-3^**′**^)**
Primers and probes screening assay (E gene assay)	E gene	E_Sarbeco_F1 E_Sarbeco_R2 E_Sarbeco_P1	ACAGGTACGTTAATAGTTAATAGCGT ATATTGCAGCAGTACGCACACA FAM-ACACTAGCCATCCTTACTGCGCTTCG -BHQ
	RNaseP gene (Internal control)	RNaseP Forward	AGATTTGGACCTGCGAGCG
		RNaseP Reverse	GAGCGGCTGTCTCCACAAGT
		RNaseP Probe	FAM-TTCTGACCTGAAGGCTCTGCGCG-BHQ
Primers and probes confirmatory assay (RdRp and ORF gene assay)	RdRp	RdRP_SARSr-F2 RdRP_SARSr-R1 RdRP_SARSr-P2 Specific for Wuhan-CoV	GTGARATGGTCATGTGTGGCGG CARATGTTAAASACACTATTAGCATA FAM-CAGGTGGAACCTCATCAGGAGATGC-QSY
	HKU ORF gene	HKU-ORF1b-nsp14F	TGGGGYTTTACRGGTAACCT′
		HKU-ORF1b-nsp14 R	AACRCGCTTAACAAAGCACTC
		HKU-ORF1b-nsp14 P	FAM-TAGTTGTGATGCWATCATGACTAG-QSY

ICMR also recommends use of FDA approved Cartridge-Based Nucleic Acid Amplification Test (CBNAAT) using Cepheid Xpert Xpress SARS-CoV2 for use under an emergency use authorization (EUA) only. CBNAAT test should be run under biosafety 2 level (BSL-2) conditions and with appropriate biosafety precautions. This test detects E gene and also the SARS-CoV-2 specific N2 region of the N gene.

RT-PCR tests involve fairly complex steps and take nearly 24–48 h for generating the results. RT-PCR tests also require trained personnel and well-equipped modern laboratories with BSL-2 set up for their use. Advent of rapid nucleic-acid-detection-based tests appear to accelerate the COVID-19 diagnosis in India. One such test is TruNat, an indigenous testing developed originally for tuberculosis, has been explored and is now being used for COVID-19 testing in India. TruNat beta CoV test, a microchip-based real-time PCR assay, runs on TruNat machines with a very short test duration of 1 h. The Truelab workstation (Molbio Diagnostics, India) includes sample preparation, an RNA extraction system, an RT-PCR machine, and disposable kit components. It is a chip-based, real-time quantitative PCR system that is portable, battery-operated, fully automated, and weighs around 3 kg. This system can be used in remote areas and has network data transfer ability and an automated reporting system. The advantage of TruNAT is that the virus is lysed during the testing process, minimizing the risk of infection and contamination by the virus. The TruNat test is a semi-quantitative real-time PCR assay that has two steps. Step 1 is and E gene screening assay. All negatives are to be considered as true negatives. All positive samples should be subjected to confirmation by Step 2 RdRp gene confirmatory assay. All samples that test positive by this assay are considered as true positive. Further RT-PCR-based confirmation is not required for samples that are positive after Step 2 of the TruNat assay (19).

On May 19, 2020, ICMR declared TruNat system as a comprehensive assay for screening and confirmation of COVID-19 cases in India. State health departments have been working with the National Tuberculosis Elimination Programme (NTEP) to establish TruNAT test for COVID-19 diagnosis. TruNAT test is promising especially in areas/districts where modern laboratories are not available. ICMR has scaled up COVID-19 testing laboratories in partnership with DST, DBT, ICAR, CSIR, DRDO, MHRD, medical colleges, and private laboratories. As of May 30, 2020, there has been a total of 669 COVID-19 testing labs in India, including 466 government laboratories and 203 private laboratories. Of these, 480 labs are using RT-PCR-based tests, 134 labs are using TruNat tests, and 55 labs are using CBNAAT-based COVID-19 tests (20).

### Pooling of Samples for Surveillance Purposes

COVID-19 cases are increasing exponentially in India. Surveillance of migrant workers and international passengers in institutional quarantine facilities is of utmost importance to understand the disease status and ultimately containing the disease spread. Hence, it becomes crucially important to scale-up and increase the numbers of tests conducted by laboratories. Because positivity rates in suspected cases are still low, pooling of samples for screening seem to be a viable option with substantial time and cost saving benefits. A pooled testing algorithm involves the PCR screening of a specimen pool comprising multiple identified individual specimens, followed by individual specimen testing only if a pooled sample tests positive. All individual samples in a negative pool are regarded as negative, and the test result requires it to be conveyed to the concerned quarantine facility within 24 h (21).

### Limitations of NAAT

Various factors contribute to a false negative result in NAAT. Factors such as poor quality of the specimen, specimen with little patient material, specimen collected late or very early in the infection, improperly handled and shipped specimens, and inherent technical reasons such as virus mutation will hamper the results of RT-PCR-based testing. WHO recommends that each NAAT run should include both external and internal controls, and they encourage laboratories to participate in external quality assessment schemes (14).

### Serology-Based Antibody Testing

Antibodies in the blood are detected when the body is responding to a specific infection, like COVID-19. IgM is one of the first types of antibodies to be produced and is the most useful for determining recent infection. Antibodies may not be detected in the early days of an infection when the body's adaptive immune response is still building. In a study, the presence of IgM antibodies for SARS-CoV-2 has been observed to range from 7 to 10 days after the onset of symptoms (22). Therefore, serology-based antibody testing is not used as the sole basis to diagnose or exclude SARS-CoV-2 infections. However, antibody testing could play a role in the fight against COVID-19 by helping health-care professionals identify individuals have developed an adaptive immune response to SARS-CoV-2. In addition, these test results can help in identifying individuals who can donate convalescent plasma, which may serve as a possible treatment for those who are seriously ill from COVID-19.

ICMR recommends using a rapid antibody test as a surveillance tool for epidemiological purposes in hot spot areas and in such areas where cases have not emerged so far. ICMR-NIV has successfully developed and validated anti-SARS-CoV-2 human IgG ELISA test kit for antibody detection of COVID-19. In an external validation, the sensitivity and specificity IgG ELISA kit was found to be of 98.7 and 100%, respectively. The ELISA test has the advantage of processing 90 samples together in a single run of two-and-a-half hours and also has minimal biosafety and biosecurity requirements as compared to the real-time RT-PCR test. The IgG ELISA test has been proposed to be used for surveillance of the population exposed to SARS-CoV-2 coronavirus infection (23).

## Antigen Testing

Antigen-based rapid tests detects the presence of SARS-CoV-2 antigens such as the nucleocapsid (N) protein and the S1 or S2 domains of the spike (S) protein. The antigen(s) detected are expressed only when the virus is actively replicating and when antigen is present in sufficient concentrations and run a higher risk of not being able to detect viral material from a swab, and are prone to produce false negative diagnosis. Therefore, antigen tests are best used to identify acute or early infection, especially in settings where a rapid test turnaround time is required. The sensitivity of antigen tests reportedly varies from 34 to 80%, which means there could be nearly 50% of false negative results, depending on the group of patients tested (3, 24). Hence, it is important for clinicians and laboratory personnel to understand the analytic performance characteristics of antigen test assays, including sensitivity, specificity, and positive and negative predictive values. As of January 6, 2021, ICMR has validated 63 antigen-based Rapid Test Kits approved for use in India. Details of these kits can be accessed on https://www.icmr.gov.in/pdf/covid/kits/List_of_rapid_antigen_kits_17022021.pdf. Antigen-based rapid tests that are US-FDA approved can be used directly after due marketing approval from DCGI.

In September 2020, ICMR issued advisory recommending the use of a rapid antigen test (RAT) as an initial test for surveillance in containment zones and for screening at points of entry. Negative RAT result should always to be confirmed by RT-PCR/TruNat/CBNAAT. Whereas in noncontainment zones and in hospital settings, RT-PCR/TruNat/CBNAAT is recommended for initial screening and followed by RAT (25). The clinical performance of RATs largely depends on the circumstances in which they are used and perform best when the viral load of the sample is generally highest. In December 2020, the CDC issued interim guidelines on the use of antigen tests for screening in high-risk congregate settings in which repeat testing could quickly identify persons with a SARS-CoV-2 infection to inform infection prevention and control measures. Laboratory professionals who perform antigen tests need to understand the factors that affect the accuracy of antigen testing, as described in this guidance (26).

## Conclusion

COVID-19 is a global pandemic and currently is the most dreadful viral disease faced by the global community. In many countries including India, the government is making enormous efforts to contain the spread of virus by implementing measures like countrywide shutdown of public places, isolating infected individuals, treatment, primary, and secondary contact tracing, decontaminating and sealing down the infected zones, etc. The ICMR, the apex body in India for the biomedical research, is at the forefront of the battle against COVID-19. ICMR is issuing advisories and appropriate guidelines regularly for tackling operational challenges, including logistics of sampling materials, testing kits, validation of kits, developing protocols, etc.

Laboratory diagnosis of SARS-CoV-2 is extremely important in the disease and outbreak management. Development of rapid point-of-care tests with better sensitivity and specificity is the critical need of the hour because this will help accurately diagnose and aid in containing the spread of SARS-CoV-2 infection. Early detection of infection greatly enhances implementation of specific public health intervention, such as infection control, environmental decontamination, and the closure of specific high-risk zones. Many aspects of the COVID-19 virus and disease are still being explored. A better understanding of viral dynamics, immunological response, duration of viral shedding etc. will help decide optimal type and timing of clinical material to be sampled for molecular testing and most importantly help decide on treatment modalities. Large-scale sequencing of the SARS-CoV-2 genome isolated from affected population across the world needs to be carried to monitor mutations that might affect performance of molecular tests. Creation of genome repositories and open-source genetic databases for use by global researchers is clearly the way forward to manage the COVID-19 outbreak and accelerate vaccine development.

## Author Contributions

KK: literature review, concept outline development, and drafting of manuscript. RN: critical review. DH: supervision. VS: literature review. SM: concept development and guidance. All authors contributed to the article and approved the submitted version.

## Conflict of Interest

All authors are affiliated to Department of Translational Medicine and Therapeutics, HealthCare Global Enterprises Ltd. (HCG), Bangalore, India. The authors declare that the research was conducted in the absence of any commercial or financial relationships that could be construed as a potential conflict of interest.

## References

[1] World Health Organization. Available online at: https://covid19.who.int/ (accessed May 14, 2020).

[2] Centers for Disease Prevention and Control (CDC). Interim Guidelines for Collecting, Handling, and Testing Clinical Specimens from Persons for Coronavirus Disease 2019 (COVID-19). (2020). Available online at: https://www.cdc.gov/coronavirus/2019-nCoV/lab/guidelines-clinical-specimens.html (accessed 14 May, 2020).

[3] Word Health Organization. Advice on the Use of Point-of-Care Immunodiagnostic Tests for COVID-19. Available online at: https://www.who.int/news-room/commentaries/detail/advice-on-the-use-of-point-of-care-immunodiagnostic-tests-for-covid-19 (accessed May 14, 2020).

[4] Stadler K Masignani V Eickmann M. SARS–beginning to understand a new virus. Nat Rev Microbiol. (2003) 1:209–18. 10.1038/nrmicro775 15035025 PMC7097337

[5] Wu A Peng Y Huang B Ding X Wang X Niu P. Commentary genome composition and divergence of the novel coronavirus (2019-nCoV) originating in China. Cell Host Microbe. (2020) 27:325. 10.1016/j.chom.2020.02.001 32035028 PMC7154514

[6] Zhou P Yang XL Wang XG Hu B Zhang L Zhang W. A pneumonia outbreak associated with a new coronavirus of probable bat origin. Nature. (2020) 579:270–3. 10.1038/s41586-020-2012-7 32015507 PMC7095418

[7] Lu R Zhao X Li J Niu P Yang B Wu H. Genomic characterisation and epidemiology of 2019 novel coronavirus: implications for virus origins and receptor binding. Lancet. (2020) 395:565–74. 10.1016/S0140-6736(20)30251-8 32007145 PMC7159086

[8] Subissi L Posthuma CC Collet A Zevenhoven-Dobbe JC Gorbalenya AE Decroly E. One severe acute respiratory syndrome coronavirus protein complex integrates processive RNA polymerase and exonuclease activities. Proc Natl Acad Sci U.S.A. (2014) 111:E3900–E9. 10.1073/pnas.1323705111 25197083 PMC4169972

[9] Wang M Cao R Zhang L Yang X Liu J Xu M. Remdesivir and chloroquine effectively inhibit the recently emerged novel coronavirus (2019-nCoV) *in vitro*. Cell Res. (2020) 30:269–71. 10.1038/s41422-020-0282-0 32020029 PMC7054408

[10] Fehr AR Perlman S. Coronaviruses: an overview of their replication and pathogenesis. Methods Mol. Biol. (2015) 1282:1–23. 10.1007/978-1-4939-2438-7_1 25720466 PMC4369385

[11] Specimen Collection Packaging and Transport Guidelines for 2019 Novel Coronavirus (2019-nCoV). Available online at: https://niv.co.in/SOP_Specimen_Collection_2019-nCoV.pdf (accessed May 14, 2020).

[12] ICMR. Specimen Referral Form for COVID-19 (SARS-CoV2). Available online at: https://www.icmr.gov.in/pdf/covid/labs/Revised_SRF_Form_16122020_1.pdf (accessed May 29, 2020)

[13] Zhang W Du RH Li B Zheng XS Yang XL Hu B. Molecular and serological investigation of 2019-nCoV infected patients: implication of multiple shedding routes. Emerg Microbes Infect. (2020) 9:386–9. 10.1080/22221751.2020.1729071 32065057 PMC7048229

[14] WHO. Laboratory Testing for 2019 Novel Coronavirus (2019-nCoV) in Suspected Human Cases. Available online at: https://www.who.int/publications-detail/laboratory-testing-for-2019-novel-coronavirus-in-suspected-human-cases-20200117 (accessed May 14, 2020).

[15] Performance Evaluation of Commercial Kits for Real Time PCR for Covid by ICMR Identified Validation Centres. Available online at: https://www.icmr.gov.in/pdf/covid/kits/RT_PCR_Tests_Kits_Evaluation_Summ_12022021.pdf (accessed May 29, 2020).

[16] ICMR. Guidelines for COVID_19 Testing in Private Laboratories in India. Available online at: https://www.icmr.gov.in/pdf/covid/labs/Notification_ICMR_Guidelines_Private_Laboratories.pdf (Accessed May 14, 2020).

[17] ICMR. National Institute of Virology (ICMR-NIV) COVID-19 Screening Assay. Available online at: https://www.icmr.gov.in/pdf/covid/labs/1_SOP_for_First_Line_Screening_Assay_for_2019_nCoV.pdf (accessed May 29, 2020).

[18] ICMR. National Institute of Virology (ICMR-NIV) COVID-19 Confirmatory Assay. Available online at: https://www.icmr.gov.in/pdf/covid/labs/2_SOP_for_Confirmatory_Assay_for_2019_nCoV.pdf (accessed May 29, 2020)

[19] ICMR. Revised Guidelines for TrueNat testing for COVID-19. Available online at: https://www.icmr.gov.in/pdf/covid/labs/Revised_Guidelines_TrueNat_Testing_19052020.pdf (accessed May 29, 2020).

[20] COVID-19 Testing Laboratories Reporting to ICMR. Available online at: https://www.icmr.gov.in/ctestlab.html (accessed May 30, 2020).

[21] Guideline for RT-PCR Based Pooled Sampling for Migrants/Returnees from Abroad/Green Zones. Available online at: https://www.mohfw.gov.in/pdf/GuidelineforrtPCRbasedpooledsamplingFinal.pdf (accessed May 25, 2020).

[22] To KK Tsang OT Leung WS Tam AR Wu TC Lung DC. Temporal profiles of viral load in posterior oropharyngeal saliva samples and serum antibody responses during infection by SARS-CoV-2: an observational cohort study. Lancet Infect Dis. (2020) 20:565–74. 10.1016/S1473-3099(20)30196-1 32213337 PMC7158907

[23] Indian Council of Medical Research (ICMR) and National Institute of Virology (NIV). Pune Develops and Validates the Indigenous IgG ELISA Test “COVID KAVACH ELISA” for Antibody Detection for COVID-19. Available online at: https://www.icmr.gov.in/pdf/press_realease_files/ICMR_PressRelease_14052020.pdf (accessed May 30, 2020).

[24] Bruning AHL Leeflang MMG Vos JMBW Spijker R de Jong MD Wolthers KC. Rapid tests for influenza, respiratory syncytial virus, and other respiratory viruses: a systematic review and meta-analysis. Clin Infect Dis. (2017) 65:1026–32. 10.1093/cid/cix461 28520858 PMC7108103

[25] ICMR. Advisory on Strategy for COVID-19 Testing in India (Version 6). Available online at: https://www.icmr.gov.in/pdf/covid/strategy/Testing_Strategy_v6_04092020.pdf (accessed January 15, 2021).

[26] Centers for Disease Control Prevention: Interim Guidance for Antigen Testing for SARS-CoV-2. Available online at: https://www.cdc.gov/coronavirus/2019-ncov/lab/resources/antigen-tests-guidelines.html (accessed January 15, 2021).

